# Transit and Fairness: Exploring Spatial Equity in Accra's Public Transport System

**DOI:** 10.1016/j.aftran.2024.100012

**Published:** 2024-01-01

**Authors:** Elvis Kyere-Gyeabour, Aruna Sivakumar, Samuel Agyei-Mensah

**Affiliations:** a Department of Geography and Resource Development, University of Ghana, Ghana; b Department of Civil and Environmental Engineering, Imperial College London, United Kingdom

**Keywords:** public transport, accessibility, spatial equity, Geographic Information System (GIS), Accra, Ghana

## Abstract

Globally, millions of individuals access services and opportunities on a daily basis using different modes of motorized and non-motorized transportation. However, in the global south, the role of public transport in providing access to services is relatively under-researched due to non-functional public transport services and poor infrastructure. This paper uses data from the Greater Accra Metropolitan Area (GAMA) to empirically contribute to the discourse on how public transport availability varies across different residential locations and assess if there is equitable access to Public Transport Infrastructure and Services (PTIS) across urban and peri-urban areas. A household questionnaire survey was designed to collect data on public transport access based on socioeconomic, socio-cultural, personal preferences/experiences and residential location in the Greater Accra Metropolitan Area (GAMA). A sample size of 1340 respondents, consisting of males and females between the ages of 18 and 70 residing in GAMA, was achieved. The paper also used data from the public and open databases. The Three-Step Floating Catchment Area (3SFCA) and geospatial methods were used to estimate spatial accessibility.

The study found a significantly high disparity in accessibility to public transport in the Greater Accra Metropolitan Area (GAMA). There is also significant spatial inequality in the level of access to Public Transport Infrastructure and Services (PTIS) in GAMA. The study revealed that the planning and provisioning of public transport infrastructure in GAMA has left areas with inequitable access to transport services. There is the need for increased investment in public transport infrastructure in EAs where Public Transport Infrastructure and Services (PTIS) were found to be very low or low in GAMA.

## 1 Introduction

Globally, millions of individuals access services and opportunities on a daily basis using different modes of motorized and non-motorized transportation (Langford et al., 2022). Spatial accessibility of transport infrastructure and services needed to meet the travel demand of the population is an indicator of transport spatial equity (Boisjoly et al., 2020; Cui et al., 2019; Geurs & Ritsema van Eck, 2001; Geurs & Van Wee, 2004; Litman, 2017; Xia et al., 2019). Some factors that are observed to contribute to public transport equity are short travel distances, faster transport modes, updated travel information and affordable costs (Lucas et al., 2016).

The increasing urban population in developing countries has made the demand for public transport services a need for individuals of all socioeconomic classes and vital to the achieving of the Sustainable Development Goals (SDG) (Guterres, 2017; Sachs et al., 2021; Sachs et al., 2017; United Nations Population Council, 2017; World Bank Group, 2017, 2020). There is often inadequate public transport infrastructure and services, resulting in unmet travel demand and overutilization of infrastructure beyond initial design capacity, especially in developing countries (Acheampong & Asabere, 2022; Campbell et al., 2019; du Preez et al., 2019; Dumedah et al., 2023; Falchetta et al., 2021; Saddier et al., 2017).

Public transport spatial equity focuses on the equal distribution of public transport infrastructure and services to all individuals in a selected location, irrespective of their socioeconomic class. The lack of non-functional public transport service results in high motorization of global south cities (Armah et al., 2010; Garcia et al., 2021). High motorization in Greater Accra Metropolitan Assembly (GAMA), like in other global south cities, results in increasing traffic congestion (see Agyapong and Ojo (2018)), noise pollution (see Khan et al. (2018); Okokon et al. (2018)) and high traffic-related air pollution (see J. Wang et al. (2022)) with detrimental health effects (Khreis et al., 2016).

A functional public transport system is essential for urban areas and affects various facets of an individual's life, especially for the less privileged (B. Wang et al., 2022). Global South cities often have private bus services and unions providing commercial transport services to the public, referred to as paratransit or other local names such as ‘Trotro’ in Accra, or ‘Danfo’ in Lagos and ‘Matatus’ in Nairobi (du Preez et al., 2019; Dumedah & Eshun, 2020; Falchetta et al., 2021; Saddier et al., 2017).

Public transport is highly under-researched in the global south due to non-functional public transport services and poor infrastructure. There has been some research on paratransit in the global south (du Preez et al. (2019); Dumedah et al. (2023); Falchetta et al. (2021); Saddier et al. (2017)). However, the nexus of public transport spatial accessibility and equity in the Greater Accra Metropolitan Area (GAMA) has received little attention. Previous studies by Saddier et al. (2017) focused on paratransit service reliability in Accra. Sadier provided important insight into the state of public transport in the Greater Accra Metropolitan Area (GAMA).

The paper aims to empirically contribute to the discourse on how public transport availability varies across different residential locations and assess if there is equitable access to Public Transport Infrastructure and Services (PTIS), with the Greater Accra Metropolitan Area (GAMA) as the empirical context. Are individuals living in GAMA's peri-urban areas travelling longer to access PTIS than others living in urban areas of GAMA?

The paper identifies the current travel distance of residents across different residential locations in the Greater Accra Metropolitan Assembly (GAMA), and evaluates the difference in the potential and actual distance individuals travel to reach Public Transport Access Points (PTAP) when meeting their mobility needs. Also, the spatial equity of PTIS in urban and peri-urban areas in GAMA is assessed. The analysis is based on a city-wide household survey of 1340 Greater Accra Metropolitan Area (GAMA) respondents conducted to identify residents' PTAPS and how PTIS impacts their mobility. The paper assesses if the local population, especially in peri-urban areas, has fair access to PTIS. The results present empirical evidence of how people in peri-urban areas in GAMA travel a longer distance to reach PTIS, resulting in high spatial inequality across GAMA.

## 2 Literature Review

### 2.1 Public Transport Infrastructure and Services

The high population density, fast urbanization and worsening transport situation make it essential for transport research to be always updated to provide needed policy inputs. There have been several studies focused on travel behaviour in Accra by scholars, including Armah et al. (2010), Abane (1993, 2011), Møller-Jensen et al. (2012), Oteng-Ababio and Agyemang (2015), Birago et al. (2017), Agyemang (2017) and Agyapong and Ojo (2018). These studies have not explored how the urbanization of the Greater Accra Metropolitan Area and the changing transportation landscape impact accessibility to transport and travel patterns.

Over the years, poor transport infrastructure, lack of functional public mass transport, lack of timely travel information, urban sprawl, and poor policy implementation are the major contributory factors to public transport inequality in Accra (Esson et al., 2016; Korean International Cooperation Agency & Ministry of Transport, 2016; Møller-Jensen et al., 2012).

These growing urban transportation problems in Accra may be affecting certain groups of individuals more than others. Equitable access to public transport is essential for vulnerable and poor socioeconomic households to improve their quality of life and well-being.

Previous studies on transport in Ghana have focused on travel behaviour, modes, patterns, and mobility options in the Greater Accra Metropolitan Area (GAMA) (Esson et al., 2016; Møller-Jensen et al., 2012). Also, previous research has examined access to markets, jobs, and economic centres (Abane, 2011; Agyapong & Ojo, 2018; Agyemang, 2017).

The absence of a functional public mass transport service in GAMA has driven changes in transport modes, partly due to socioeconomic demand and technological advancement, with the state-run public transport system collapsing in the 1980s due to mismanagement and economic crises (Abane, 1993). The introduction of the Metro Mass Transit (MMT) in the early 2000s was not sustainable due to financial and social mismanagement (Agyemang, 2015; Birago et al., 2017; Gaisie et al., 2019). The Bus Rapid Transit (BRT) was also introduced in 2016 to address the mass public transit need (Agyemang, 2015; Gaisie et al., 2019).

However, the Accra BRT services failed to operate as a BRT due to several problems, including institutional constraints and support, lack of transport terminals, transport union agitations, non-dedicated lanes, lack of an intelligent traffic management system and cost over-runs (CitiNewsRoom, 2018; Fan & Beukes, 2021; Mudu & World Health Organization, 2021).

#### 2.1.1 Paratransit Services and Transport Modes in Accra

Paratransit in global south developing countries is characterized by their informal nature, non-fixed route and non-scheduled transportation system (du Preez et al., 2019; Dumedah et al., 2022; Falchetta et al., 2021; Saddier et al., 2017).

The major paratransit transport service in GAMA is the "trotro" different private transport unions, such as Ghana Private Road Transport Union (GPRTU) and Progressive Transport Operators Association (PROTOA) (Abane, 2011; Birago et al., 2017; Dumedah et al., 2023). There is a lack of incorporation of paratransit public transport systems in accessibility studies due to their informal nature, creating a knowledge gap over the years. Over the years, emerging modes of transport in the GAMA have included motor taxis ("Okada"), tricycle-taxi ("Pragia" or "Kumkum"), Uber, Bolt and Yango. These emerging transport modes directly influence the travel patterns and transport accessibility of individuals in different spatial locations of the GAMA.

#### 2.1.2 Spatial Accessibility and Equity of Public Transport

The concept of population potential by Stewart (1947), relating the population at a location and the distance to urban service interaction, was a fundamental pillar in the evolution of accessibility. Stewart’s concept was fundamental to Hansen (1959, p. 73) definition of accessibility as “the potential of opportunities for interaction”. Hansen (1959) views accessibility with emphasis on the spatial distribution of activities about a point and an individual’s behaviour to overcome their spatial separation. Hansen refers to land use, services and accessed activities as opportunities.

Litman (2017) defined accessibility based on two terms from Hansen (1959), using the potential of opportunities available and utilized opportunities. The work of Litman (2017) and Rode et al. (2017) emphasize two types of accessibility: potential/perceived accessibility (accessibility by design) and behavioural accessibility (accessibility based on an individual or group of individuals' behaviour). The paper operationalizes the two types of accessibility by comparing the potential and behavioural public transport availability and accessibility in urban and peri-urban areas of the GAMA.

Equity is loosely synonymized with justice and fairness, with various definitions from various disciplines (Ahmed et al., 2008; Ben-elia & Benenson, 2019; Cui et al., 2022; Di Ciommo & Shiftan, 2017). The use of travel distance, frequency, income, and travel time thresholds by Cui et al. (2019) to establish equity in their measurement of levels of accessibility for the Vancouver, Montreal area in Canada. There has been limited implementation of spatial equity in accessibility research done in Sub-Saharan Africa.

## 3 Materials and methods

### 3.1 Study Area

**Figure 1:**
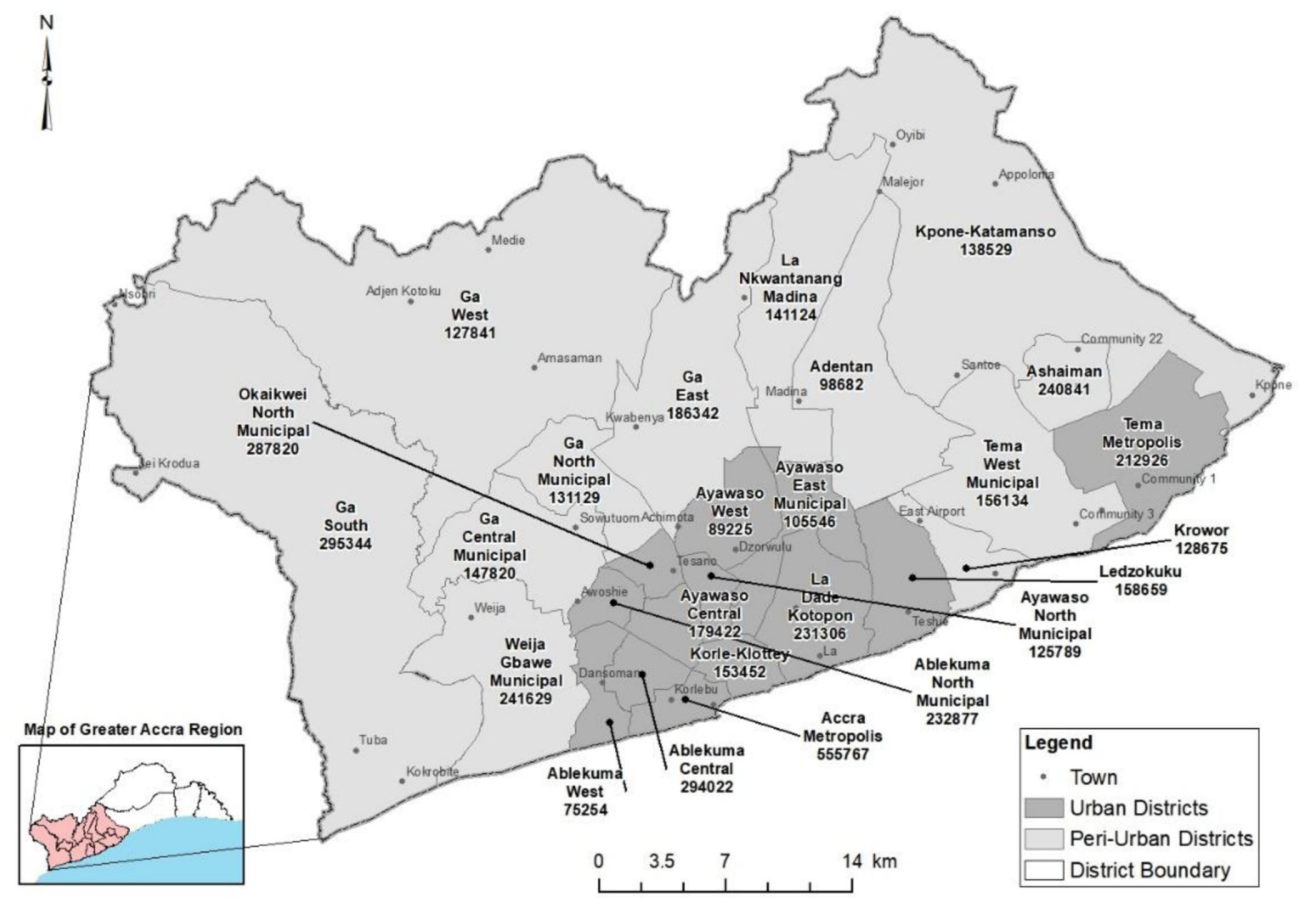
Map of the Greater Accra Metropolitan Area (GAMA). Data source: Ghana Statistical Services (2020), Author (2021)

The study was done in the Greater Accra Metropolitan Area (GAMA) in the Greater Accra Region of Ghana with a latitude of 5° 39' 7.77" N and a longitude of 0° 12' 46.79" W (Figure 1). GAMA is the urbanized and peri-urban areas of the Greater Accra Region in Ghana, comprising the Accra Metropolitan Assembly (AMA), Tema Metropolitan Assembly (TMA) and 23 other municipal assemblies (Figure 1).

As of 2020, GAMA had a population of 4.7 million residents, from 3.8 million in 2010 (Ghana Statistical Service, 2013; Ghana Statistical Services, 2020). The population projection of GAMA is estimated at 10.4 million in 2040 (Ghana Statistical Service, 2013). Greater Accra Metropolitan Area (GAMA) had a population density of 3,124 individuals per km^2^ as of 2020 (Esson et al., 2016; Ghana Statistical Service, 2013, 2022). There are 16 fully urbanized districts and nine districts with significant peri-urban areas in GAMA.

There has been uncontrolled urban expansion in GAMA, resulting in settlements without basic services and infrastructure to meet their transport needs (Owusu, 2010). This is evident in the scholarly works of Agyemang (2017); Agyemang et al. (2017), and Grant (2009).

**Figure 2:**
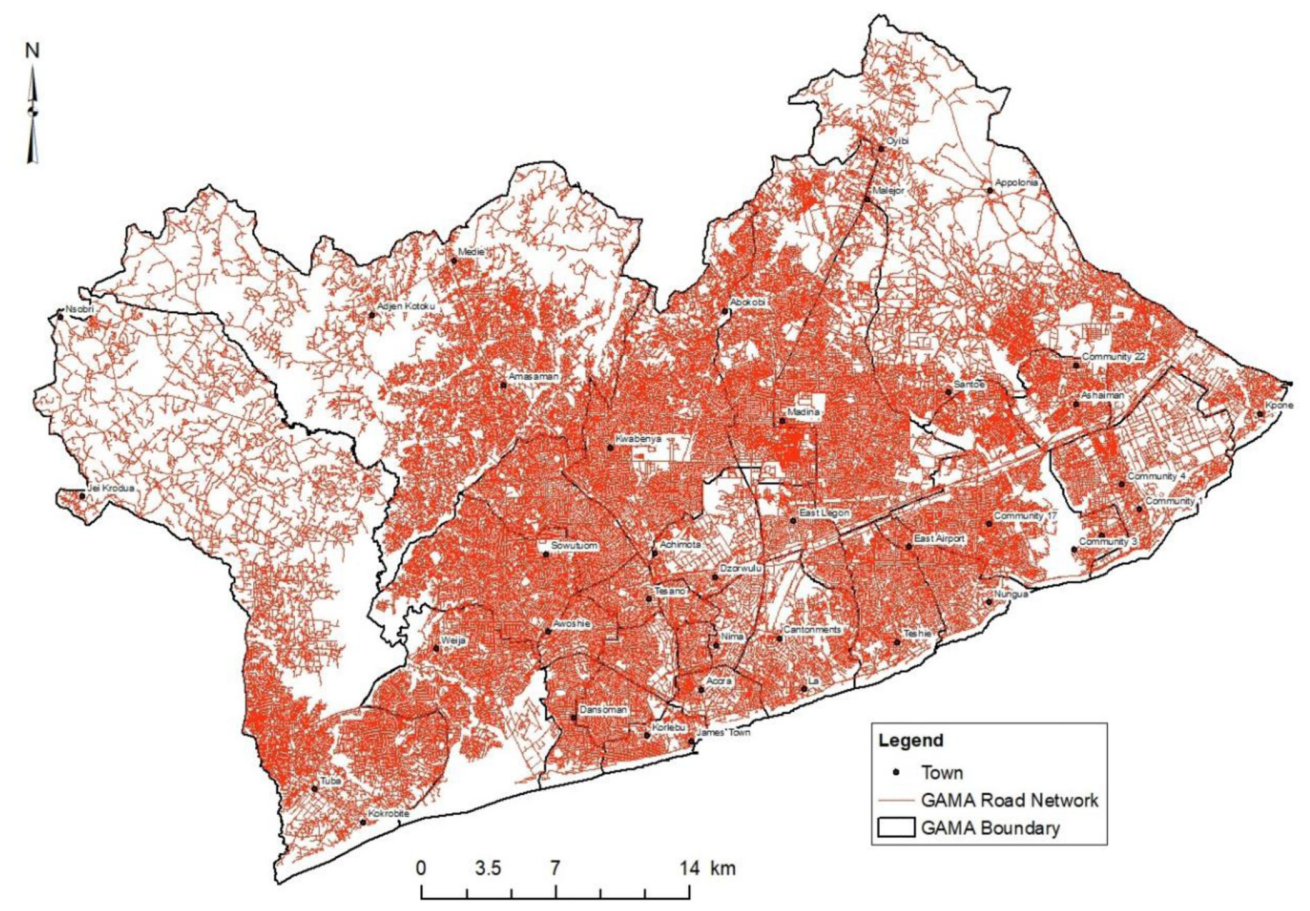
Map of the Road network for GAMA. Data source: Maxar Database (2020), Open Street Map (2021), Author (2021)

This study focuses on GAMA as located within the administrative boundaries of the Greater Accra Region as employed in other studies on GAMA (Abane, 2011; Agyapong & Ojo, 2018; Garcia et al., 2021; Tetteh et al., 2022; J. Wang et al., 2022). The road network (Figure 2) in GAMA's south and central areas are denser than areas in the outer periphery of GAMA. Despite the rapid increase in urban population and urban development of GAMA, public transport infrastructure and services have not been expanded to meet increased travel demands and high vehicular congestion (Agyapong & Ojo, 2018; Esson et al., 2016; Møller-Jensen et al., 2012).

**Figure 3:**
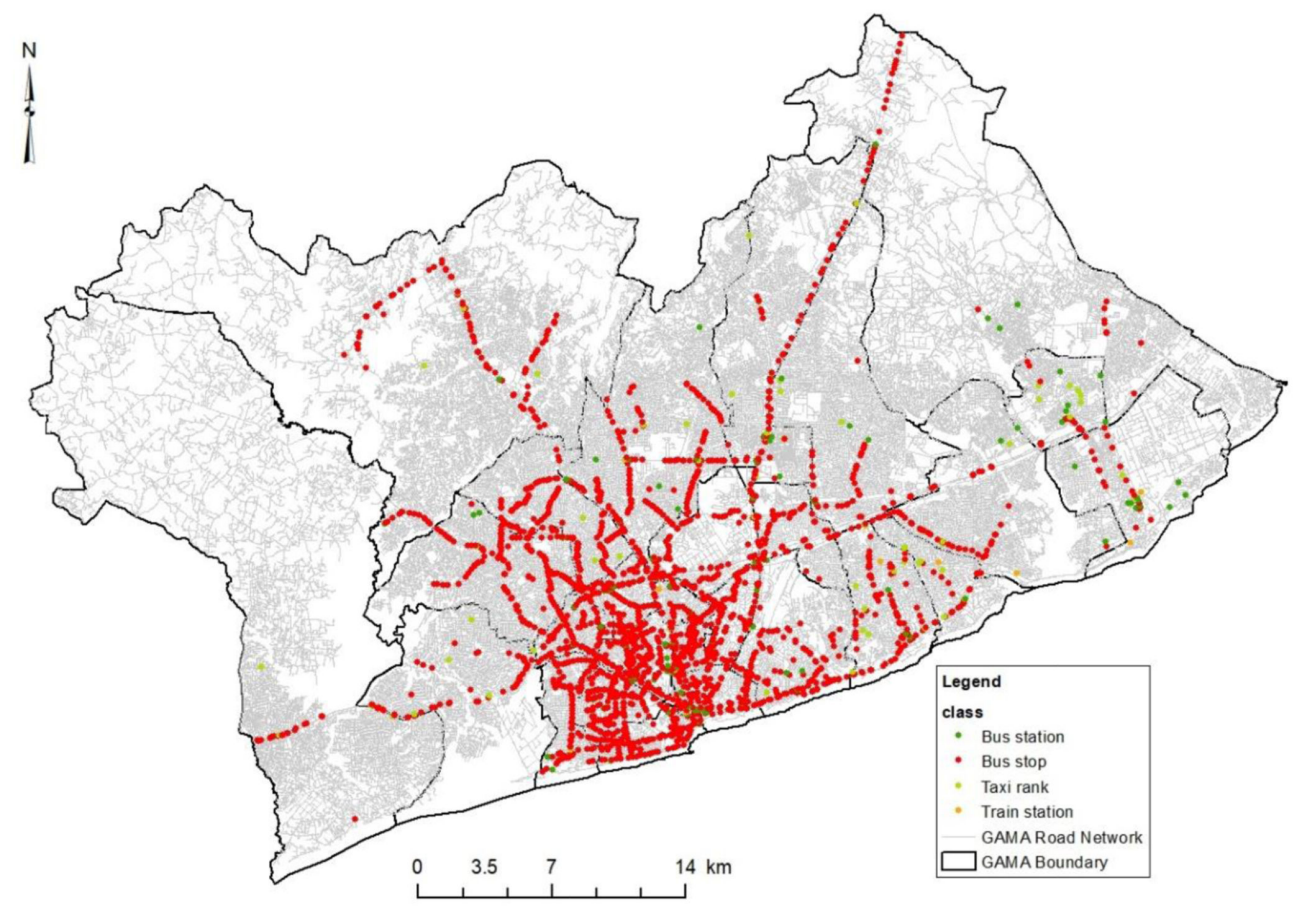
Public Transport Access Points (PTAP) in GAMA. Data source: Maxar Database (2020), Open Street Map (2021), Author (2021)

Public Transport Access Points (PTAP) data included bus stations, bus stops, taxi stations and train stations in GAMA. The PTAP is presented over the neighbourhood road network for Greater Accra Metropolitan Accra (GAMA) (Figure 3). Figure 3 shows the spatial distribution of different classes of PTAP on the road network across GAMA. Highways and major roads have a higher frequency of PTAPs compared to neighbourhood or residential streets.

### 3.2 Survey Design and Sampling Technique

The paper used a household questionnaire survey to collect data on public transport access based on socioeconomic, socio-cultural, personal preference/experiences and residential location in the Greater Accra Metropolitan Area (GAMA). The study used a grid sampling method has been employed extensively in the assessment of city-wide accessibility using the Floating Catchment Area (FCA) analysis by researchers such as Bauer and Groneberg (2016); Delamater et al. (2019); Kanuganti et al. (2016); Kiani et al. (2021); Langford et al. (2012); Ni et al. (2019); Ni et al. (2015); Tao et al. (2020); and Xia et al. (2019). Proportional sampling based on the district population of the 2020 projected population by Ghana Statistical Services (2020) was adopted, targeting a sampling error of 5% with a 95% confidence interval.

The research sampled a total of 1340 respondents consisting of males and females between the age of 18 years to 70 years residing in the 25 districts of GAMA. A multi-staged sampling technique as used in studies by Ahenkan and Aduo-Adjei (2017); Akowuah et al. (2018); Boachie (2015); Boakye et al. (2017); Nketiah-Amponsah et al. (2019). The first strata of the multi-staged sampling involved a random selection of enumeration areas (EA) within each of the 25 district, as designated by the Ghana Statistical Service (GSS) (Ghana Statistical Service, 2013).

The second multi-stage sampling strata required making a 2 km x 2 km grid of GAMA, with each grid having a unique number. The numbered 2km x 2km grid sampling frame was used to generate numbers for households selected by systematic sampling.

**Figure 4:**
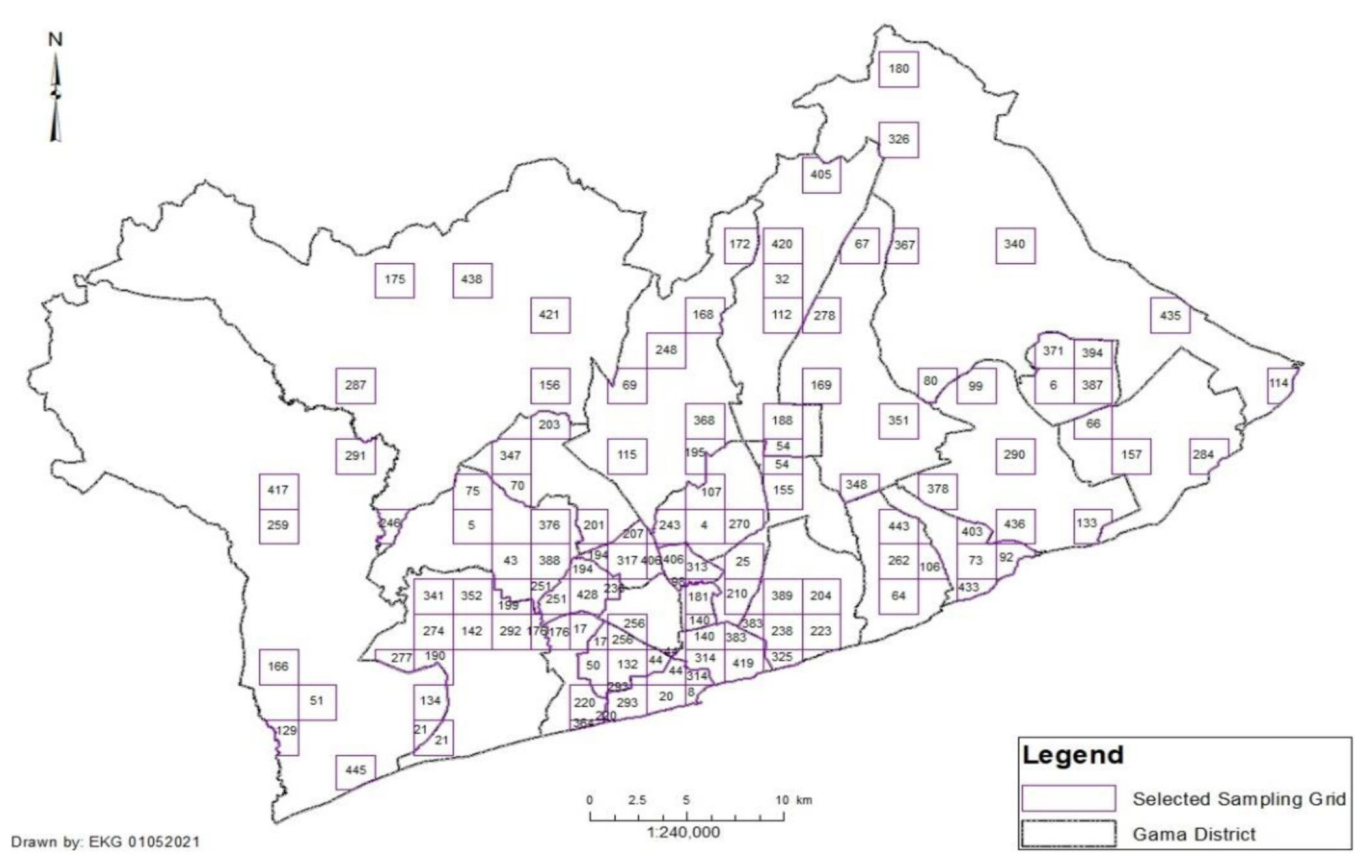
Selected EAs using sampling grid of 2km x 2km in GAMA. Data Source: Ghana Statistical Service (2013), Author (2021)

The final strata of the multi-staged stratified sampling considered the households within the randomly selected grid, as shown in Figure 4. A household was selected after every 10^th^ house and a block within the randomly selected grid in the EA. On selecting a household any available person is selected considering inclusion protocols to obtain a representative sampling size.

### 3.3 Secondary Data Sources

The paper used secondary data from the Ministry of Transportation, Ghana Statistical Service (GSS), Department of Urban Roads (DUR), Driver and Vehicle Licensing Authority (DVLA), Maxar, Google Map, Google Traffic Matrix, and Open Street Map (OSM). Ghana Statistical Service (GSS) provided population data for 2010 and 2020 and administrative boundaries for regions, districts, and Enumeration Areas (EAs) (Ghana Statistical Service, 2013, 2016; Ghana Statistical Services, 2020).

### 3.4 Data Analysis

Primary data from household survey were analysed to present descriptive analysis, crosstabulation, correlation, and Analysis of Variance (ANOVA), using IBM's SPSS version 25. All the data were geo-referenced to their respective EAs in the GAMA and stored in a geodatabase for further analysis. First, an average nearest neighbour and spatial autocorrelation analysis (Global Moran's I) was done to identify the spatial distribution of PTAPs in GAMA as employed by Amiri et al. (2021); Jana and Sar (2016); Kumar and Parida (2021); and Prasannakumar et al. (2011).

A network analysis was also done on the road network and PTAP (Figure 3) to identify the closest distance from the EA centroids for potential PTAP distance and from respondent’s locations for behavioural PTAP distance. The disparity in potential distance from EA centroid to closest PTAP was assessed for urban and peri-urban districts in GAMA. The behavioural distance of respondents from their residential locations to closest PTAP was evaluated for urban and peri-urban areas in GAMA. Public Transport Access Point (PTAP) was categorized into four classes (Figure 3) based on the Greater Accra Transport Master Plan policy document by Korean International Cooperation Agency and Ministry of Transport (2016) and the National Transport Policy of Ministry of Transportation (2008).

The Average Nearest Neighbour (ANN) analysis was used to measure the distance between each PTAP and the nearest PTAP location (Amiri et al., 2021; ESRI, 2011).

The spatial autocorrelation (Global Moran's I) was estimated by simultaneously considering PTAP locations and counts during computation. The spatial autocorrelation determined whether a feature under consideration has a random, clustered, or dispersed spatial distribution pattern (Amiri et al., 2021; ESRI, 2011).

GAMA road network (Figure 3) was also analysed using ArcGIS Network Analysis (ESRI, 2011; Wang & Xu, 2011). The network analysis model was carried out on the road and PTAP geodatabase. The network analysis model was used to determine the closest PTAP, from EA centroids and respondents’ location.

The Three-Step Floating Catchment Area (3SFCA) geospatial model was used to estimate potential public transport infrastructure and service accessibility index in GAMA (Delamater et al., 2019; Saxon et al., 2021; Subal et al., 2021; Wan et al., 2012). The 3SFCA used EA centroids, EA population, travel time or distance to a PTAP, service capacity of PTAP, number of PTAP at an EA, road network, and waiting time at PTAP.

## 4 Results and Discussion

### 4.1 Spatial Distribution of Public Transport Access Point (PTAP) in GAMA

About 92% of PTAPs were bus stops, 5% were bus stations/terminals, and train stations recorded 0.3% in GAMA (Figure 3). The Accra Metropolitan Assembly (AMA) which contains the central business district with a population of 555,767, recorded 383 PTAPs, the highest PTAP number in GAMA.

The lowest number of PTAPs, 30, was recorded in an inner-city district called Krowor Municipality. The study also found a significant difference in the average distance to a PTAP in the districts of GAMA. GAMA had an average per capita ratio of 5,770 individuals to 1 PTAP. PTAP per Capita ratio in GAMA was in the range of 770 individuals to 1 PTAP and a maximum of 10,000 individuals to 1 PTAP.

Public Transport Access Points (PTAP) showed a clustered spatial distribution in GAMA by Average Nearest Neighbour recorded a p-value of 0.0000 (Table 1) at a 0.01 alpha level. The Global Moran's I analysis of PTAPs also recorded a p-value of 0.0000 (Table 1) at a 0.01 alpha level and a clustered spatial distribution. The significant difference in the spatial distribution of PTAPs in GAMA with areas in the south-central part of GAMA, where the Central Business District (CBD) is located, with a dense concentration of PTAPs. The areas in the outer periphery of GAMA, mainly peri-urban areas, recorded few PTAPs, accounting for the significant clustering of PTAPS in GAMA.

Significant high clustering of PTAPs was found in the Accra Metropolitan Assembly (AMA), Ablekuma West, Ablekuma Central, Ayawaso East, Korle Klottey, La Dade Kotopon, and Okaikwei North municipalities (Figure 4). Certain GAMA districts had a higher number of larger PTAPs, with Ga South, Ga West, Weija-Gbawe, and Kpone-Katamanso municipalities showing more dispersed PTAPs, often resembling bus stops. However, these bus stops lack standard infrastructure, such as shelters, seating benches, ticket posts, and amenities commonly found in developed countries.

The current level of PTAP availability per capita in GAMA results in longer waiting time at terminals and bus stops as the number of people that can be served at a time is woefully inadequate. This greatly affects the accessibility of residents in GAMA to transport, education and healthcare services. The ripple effect of inaccessible transport services resulting in lack of access to basic urban services is a finding that has been made by previous studies in Accra (Agyemang et al., 2017; Esson et al., 2016; Korean International Cooperation Agency & Ministry of Transport, 2016; Møller-Jensen et al., 2012).

Residents of EAs urban areas had a denser road transport network, more PTAP and hence, a shorter distance to access public transport. Public Transport Access Points by district showed how unequal the level of availability and hence accessibility to public transport.

### 4.2 PTAP Distance Analysis in GAMA

The Public Transport Access Point (PTAP) distance analysis was done for all 6147 EAs in the GAMA to estimate the potential distance individuals travel to reach the closest PTAP. Daniels and Mulley (2013) reported that the standard distance between bus stops/stations is 400m, as the Bureau of Transport Statistics estimated. Pandey et al. (2021) found an average distance of 433m between bus stops/stations in cities in the United States of America. The Ghana Transport Policy of Ministry of Transportation (2008) does not recommend a distance threshold for the siting of bus stops, bus stations or other PTAP. The PTAP analysis adopted 400m as a standard distance for high accessibility of PTAP. The results from the city-wide survey among residents provided the actual distance travelled to reach their preferred PTAP in the GAMA

## 5 Potential PTAP Distance Analysis – Urban and Peri-urban Areas of GAMA

**Figure 5:**
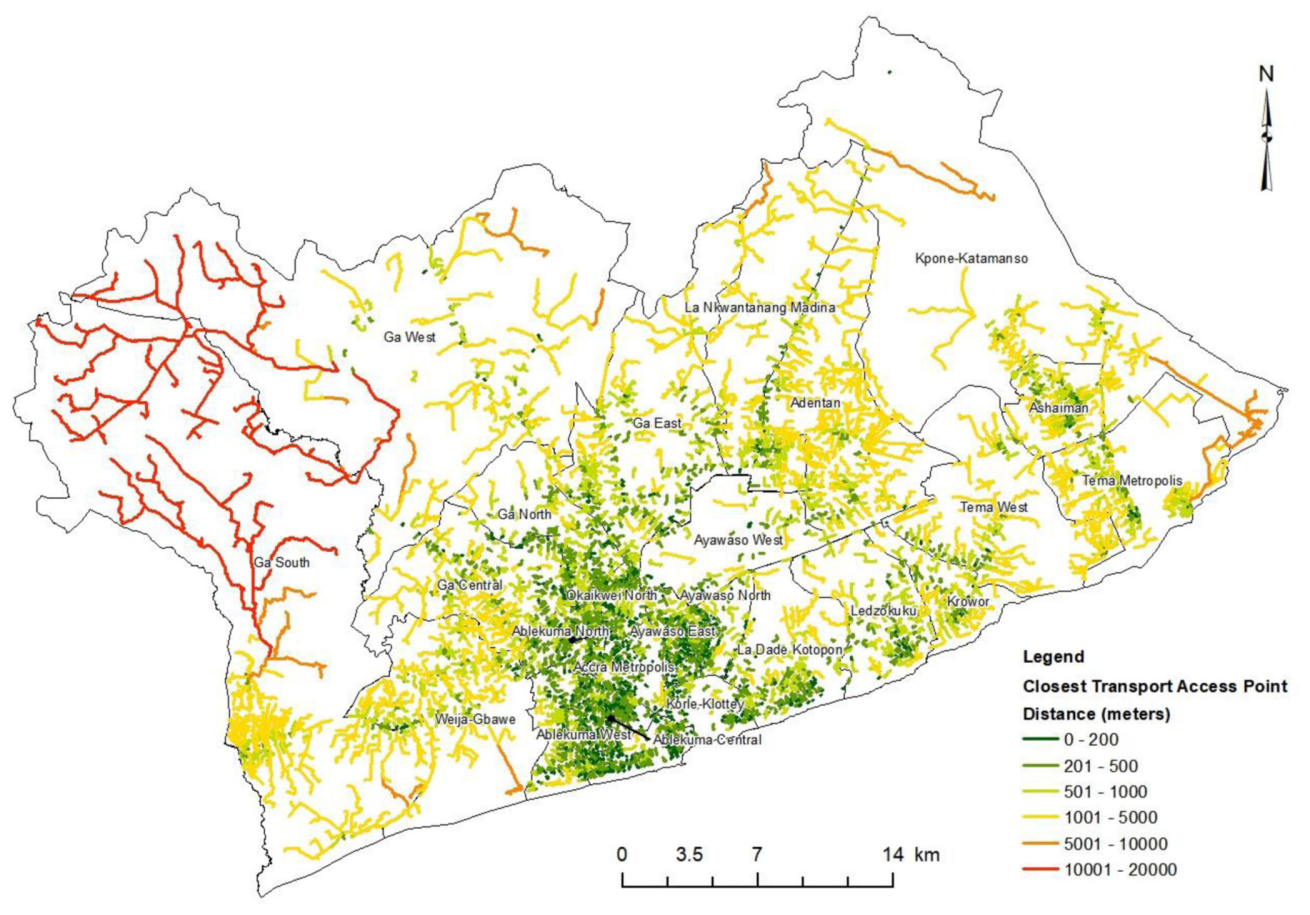
Potential Distance from EA centroid to closest Public Transport Access Point (PTAP) GAMA.

Network analysis to find the closest Public Transport Access Points (PTAPs) to households was done for all districts in Greater Accra Metropolitan Area (GAMA) shown in Figure 5. The network analysis found high disparity in physical access to PTAP in different neighbourhoods of GAMA. This can be associated with the significantly clustered spatial distribution (Table 1) of PTAP. The distance from an EA centroid to a PTAP increases as one moves away from the inner-core of Accra (Figure 5).

The north-western part of GAMA recorded longer potential distances of 10,001 – 20,000 m to the closest PTAP. The results show that neighbourhoods in the south-central part of GAMA (Figure 5) have better physical access to PTAPs than those in the outer periphery.

About 20.1% of EAs recorded a potential distance range of 0-200m to the closest PTAP in GAMA. These EAs were found in Accra Metropolitan Assembly, Ablekuma West, Ablekuma Central, Ablekuma North, Ayawaso East, Korle Klottey, Okaikwei North municipalities and parts of La Dade Kotopon municipality (Figure 5). Also, EAs that are located along major highways and paratransit routes recorded a potential distance range of 0-200m to the closest PTAP.

The potential distance range of 201-500m. 501-1,000m, 1,001-5,000m was recorded in 33%, 24.6% and 20.2% of EAs respectively. About 8.1% and 1.2% GAMA EAs recorded a potential distance of 5,001-10,000m and 10,001-20,000m to the closest PTAP respectively (Figure 5). EAs in the outer-periphery of GAMA, mostly peri-urban areas longer distance to the closest PTAP (Figure 5). The map of potential distance to the closest PTAP (Figure 5) of GAMA reinforces the unequal physical access among residents of different EAs in GAMA.

An Analysis of Variance (ANOVA) of the closest PTAP distance by districts showed a significant difference (p-value – 0.000) at a 0.05 alpha level. The significant difference hints of the disparity in the availability of PTAP meant across the different districts in GAMA. This is evidence of inequitable availability of public transport services in Accra across different levels of urbanization. Most areas with low public transport infrastructure availability are often underdeveloped and found in peri-urban areas of GAMA (Figure 5).

The level of physical access to PTAP for an individual in GAMA significantly depends on their residential location in the city. Individuals living in a fully urbanized area (Figure 5) that are at or near the inner core of GAMA have a shorter potential distance when accessing PTAP. GAMA residents living in peri-urban areas like Domeabra in the Ga South Municipality, have a longer potential distance when accessing PTAP.

This inequality in the distance when accessing public transport often results in lost opportunities and ultimately affects the quality of life of residents especially those in periurban areas.

About 64% districts in GAMA have an average distance higher than 400m to a PTAP (Table 2). About 36% of districts recorded PTAP distance of less than 400m, they were around the Central Business District (CBD) (Table 2). Accra Metropolitan Assembly a fully urbanized district recorded the shortest average distance of 216m to PTAP in GAMA.

Ga West Municipal Assembly a peri-urban district recorded the longest average PTAP distance of 5,521m.

The urban districts in GAMA all recorded less than 1-kilometer average potential distance to PTAP (Table 2). Whereas 66% of peri-urban districts recorded more an average distance of more than 1 kilometre to PTAP.

About 36% of districts recorded PTAP distance of less than 400m, they were around the Central Business District (CBD) (Table 2). Accra Metropolitan Assembly a fully urbanized district recorded the shortest average distance of 216m to PTAP in GAMA.

Ga West Municipal Assembly a peri-urban district recorded the longest average PTAP distance of 5,521m. The urban districts in GAMA all recorded less than 1-kilometer average potential distance to PTAP (Table 2). Whereas 66% of peri-urban districts recorded more an average distance of more than 1 kilometre to PTAP. The high disparity in average potential distance to PTAP between urban and peri-urban districts exposes the high spatial inaccessibility and inequality when individuals need public transport services.

Peri-urban districts are often inhabited by individuals of low social-economic status and have been found to lack not only PTAP but other basic urban services such as water and other utility services (Amankwaa, 2017; Esson et al., 2016; Tetteh et al., 2022). The findings on PTAP whiles demonstrating high spatial inaccessibility and inequality among residents of GAMA, is further heightened when the classes of PTAP are considered individually. With only 5% of PTAPs in GAMA being bus stations as shown in Figure 3, it is important for a revised transport policy to consider adopting a standard distance for bus station/terminals. By providing more bus stations within a standard distance, individuals in GAMA will have a shorter commute to PTAPs, increase the option of PTAPs to choose from and also reduce the resulting spatial inaccessibility in urban and peri-urban districts.

The study found 26.9% of respondents commuting for 0-200m to reach their preferred PTAP when accessing public transport. About 30.6% of respondents travel for 501 - 1,000m when accessing public transport in GAMA. For distance classes 5,001 – 10,000m and 10,001 – 20,000m about 8.1% and 0.3% of respondents respectively commute to reach a PTAP in GAMA. Respondents in urban EA had an average distance of 463.5 m commute when accessing a PTAP. Whereas respondents in peri-urban EA recorded an average distance of 2,765.8m when accessing PTAP. Respondents in urban EA did not travel more than 5,000m to reach a PTAP. However, respondents in peri-urban EAs travelled up to 20,000m to reach a PTAP when accessing public transport.

Respondents living in urban areas were found to travel an average 133.5m (Table 2) more than the estimated potential distance to the closest PTAP. However, respondents living in peri-urban areas had to travel an average 1,197m more than the estimated potential distance to the closest PTAP. Some respondents in La Nkwantanang Madina Municipal had to travel an average of 3,393m more than the estimated closest potential distance to a PTAP.

The road network in the fringes of GAMA was in a poor state, with fringe districts lacking tarred roads, as found by Møller-Jensen et al. (2012). The provisioning of PTAP in GAMA is often built during road construction; hence most communities that lack tarred roads also do not have adequate PTAP. This is often the case among the periphery districts in GAMA, which lack a good road network and have fewer PTAP.

This creates a double inaccessibility to transport services where a district has a poor road network and few opportunities when accessing PTAP. The disparity in potential and behavioural accessibility at a location as evident in the findings point to what type of PTAP is available in an Enumeration Area (EA). Typically, an EA that has five bus stops and one bus station will likely record high potential accessibility to PTAP mainly due to the bus stops. But the behavioural accessibility to PTAP may be lower as residents have to commute to a particular bus stop in the EA which could be farther away. The study also found resident in GAMA choosing a PTAP depending on the commute habit/past experiences, time of travel, surrounding road network, travel information, and security at the PTAP.

## 6 Potential Public Transport Infrastructure and Services (PTIS) Accessibility Index in GAMA

**Figure 6:**
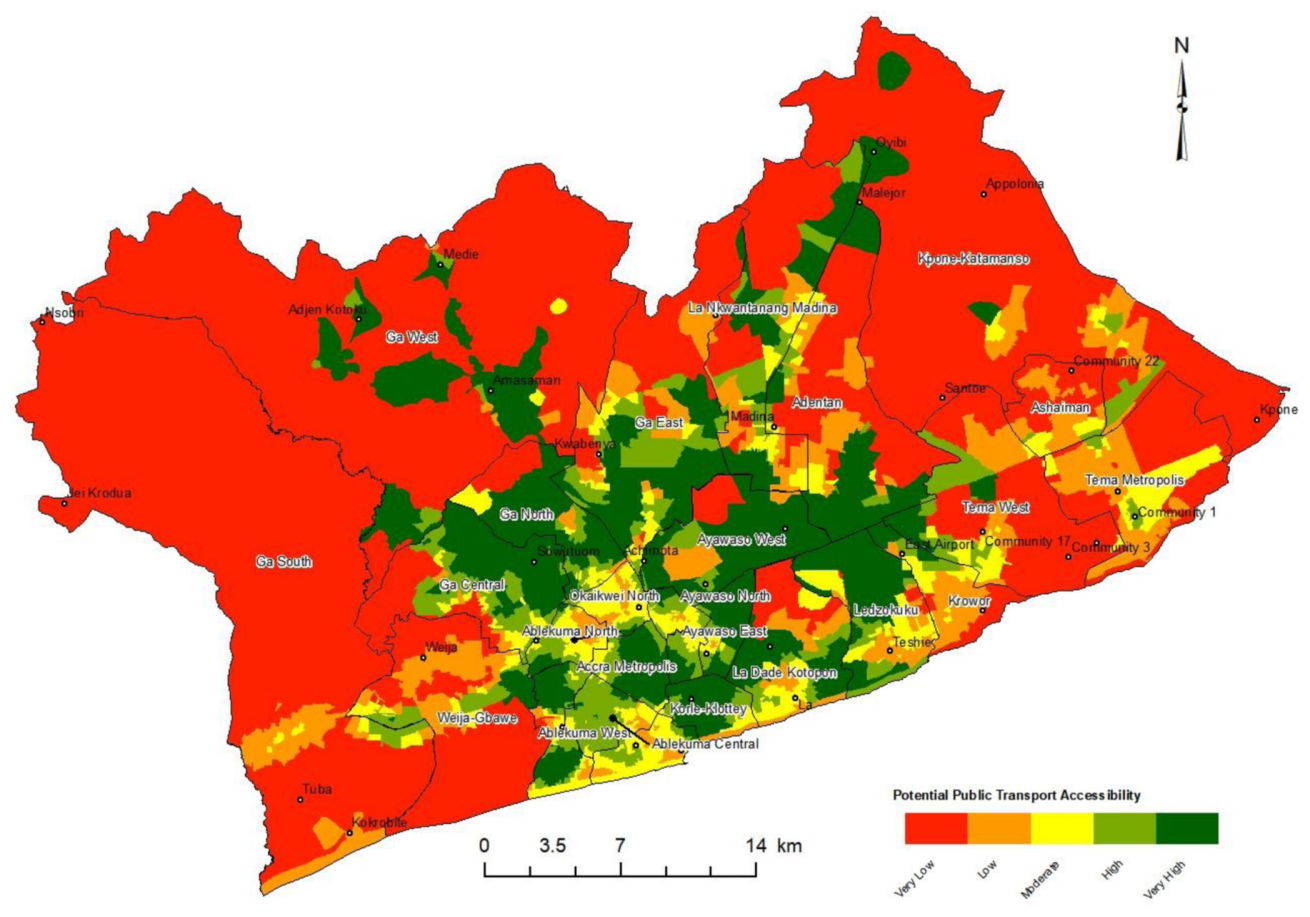
Potential public transport infrastructure accessibility index of Enumeration Areas in GAMA.

The potential accessibility index for Public Transport Infrastructure and Services (PTIS) was estimated using the Three-Step Floating Catchment Area (3SFCA) model (Figure 6). The index showed Enumeration Areas (EAs) with very high PTIS accessibility and areas with very low accessibility to PTIS.

EAs in the south-central parts, around GAMA's Central Business District (CBD), recorded very high PTIS accessibility index (Figure 6). About 0.2% of Enumeration Areas (EAs) recorded a very high public transport infrastructure accessibility index (Figure 6).

A high accessibility index was recorded by 6.6% of Enumeration Areas (EAs), and 59.1% of GAMA Enumeration Areas (EAs) also recorded a moderate accessibility index. The low accessibility index was recorded among 27.2% of GAMA's Enumeration Areas (EAs) (Figure 6). The very low public transport infrastructure accessibility index was recorded in 7% of GAMA's Enumeration Areas (EAs).

The mean potential PTIS accessibility index in GAMA was moderate accessibility index. About 24% of districts in GAMA recorded very high potential PTIS accessibility index. Low potential PTIS accessibility index was recorded in 28% of the districts in GAMA. The districts with a low average potential public transport accessibility were of low socioeconomic income status and districts located at the periphery of GAMA.

The highest average PTIS accessibility index was recorded in the Ga North Municipality (Figure 6). The lowest average PTIS accessibility score was recorded in the Ga South Municipal Assembly (Figure 6).

An Analysis of Variance (ANOVA) of PTIS accessibility index by districts showed a significant difference (p-value – 0.000) at a 0.05 alpha level. The significant difference shows the spatial inequality in the accessibility of PTIS across the different districts in GAMA.

The empirical evidence of inequitable PTIS accessibility across GAMA, points to serious impacts on residents’ quality of life especially among low income and vulnerable households.

The low public transport infrastructure accessibility can be attributed to the high rate of uncoordinated urbanization in the Greater Accra Metropolitan Area (GAMA), poor planning schemes and lack of investment in public transport infrastructure (Birago et al., 2017; Esson et al., 2016; Møller-Jensen et al., 2012; Owusu, 2010). Public transport accessibility in GAMA is strongly linked to socioeconomic status, with residents in well-connected urban areas benefiting more. High-income districts exhibit notably higher accessibility indices, highlighting the influence of economic factors on access to public transport.

High accommodation costs in areas with excellent public transport accessibility force urban poor individuals to live in districts with limited access.

Paradoxically, public transport, crucial for those with lower socioeconomic status, is primarily used by middle to low-income individuals in Accra, as high-income individuals tend to have private transportation. (Abane, 2011; Agyemang, 2017; Birago et al., 2017).

The urban locations with very high accessibility to public transport are often beyond the financial capability of middle to low-income individuals. The districts in peri-urban areas such as Ashaiman, Ga South, Kpone-Katamanso and Weija Gbawe recorded low public transport accessibility. These peri-urban areas have poor road networks, fewer PTAP, longer distance to PTAP and often a high population that require public transport. There is a growing trend where low-income residents would live in slums near the CBD to enhance their accessibility to needed opportunities (Campbell et al., 2019; Esson et al., 2016). The research findings on access to public transport adds to literature.

The paper confirms that an individual's residential location significantly impacts their access to public transport in GAMA. Peri-urban residents in Accra experience lower accessibility, potentially leading to social exclusion from essential urban services and opportunities (Adhvaryu et al., 2019; Campbell et al., 2019; Esson et al., 2016; Møller-Jensen et al., 2012). The disparity in spatial accessibility for public transport mostly affects individuals of middle to low-income status living in peri-urban areas in GAMA.

The Sustainable Development Goal 11, Target 11.2 is aimed at providing safe, affordable accessible and sustainable transport systems for all in cities (Guterres, 2017; United Nations Ghana, 2020). The potential public transport infrastructure and service accessibility index (Figure 6) is an empirical baseline for public transport infrastructure and service accessibility measure in GAMA. The SDG 11, Target 11.2 assessment for Ghana had no data as reported by Sachs et al. (2021). This shows the lack of attention given to the state of public transport services and how it affects quality of life in GAMA and Ghana as a whole.

Achieving SDG Goal 11 in Accra necessitates addressing public transport inaccessibility in peri-urban and urban areas with limited infrastructure, involving coordinated peri-urban development and effective policy implementation to enhance transport equality and quality of life in the Greater Accra Metropolitan Area (GAMA).

## 7 Conclusions

The paper found a significantly high disparity in accessibility to public transport in the Greater Accra Metropolitan Area (GAMA). There is also significant spatial inequality in the level of access to Public Transport Infrastructure and Services (PTIS) in GAMA. The study provided empirical evidence of the spatial inequity in GAMA providing public transport infrastructure and services. The study also highlighted the high population ratio to public transport infrastructure and services; and the negative impact on individuals of varying socio-economic statuses and residential locations in GAMA.

There was significant spatial clustering of public transport infrastructure around the CBD with areas near the periphery of GAMA having fewer PTAP. GAMA had an average per capita ratio of 5,770 individuals to 1 Transport Access Point. The study also found significant difference in the average distance to a transport access point in the districts of GAMA. The difference showed the huge disparity in the availability and accessibility of public transport infrastructure and services in urban and peri-urban areas in GAMA. The paper reveals the unplanned expansion of the city’s urbanization especially in peri-urban areas is evident by the lack of basic urban services including transport.

The paper presents empirical evidence of spatial inequity in public transport infrastructure and services in GAMA, emphasizing the disproportionate population-to-infrastructure ratio affecting individuals across different socio-economic statuses and residential areas. It underscores the importance of addressing this issue for achieving Sustainable Development Goal (SDG) eleven, highlighting the need for immediate policy interventions to rectify spatial inequality and improve accessibility.

## 8 Recommendations

The study recommends a population-integrated driven provisioning of public transport services in GAMA to ensure transport demand is adequately addressed and increase public transport coverage. This can be done by adopting the 400m standard for the provision of PTAP such as bus station/terminals in districts with low spatial accessibility especially in peri-urban districts.

There is the need for increased investment in public transport infrastructure like building and maintaining roads, bus station, pedestrian pathways, and bike lanes in EAs where Public Transport Infrastructure and Services were found to be very low or low in GAMA.

## Figures and Tables

**Table 1:** Spatial Distribution of Public Transport Access Points in GAMA

Spatial distribution Analysis	Ratio/Index	z-score	p-value	Alpha	Remarks
Average Nearest Neighbour	0.3227	-68.0206	0.0000	0.01	Clustered
Global Moran's I	0.0679	7.9699	0.0000	0.01	Clustered

**Table 2:** Average Potential and Behavioural Distance to the closest Public Transport Access Point (PTAP) in GAMA

District Assembly	Mean Potential PTAP (m)	Mean Behavioural PTAP (m)	Difference Potential and Behavioural (m)	Standard Distance (m)
**Urban Districts**				
Accra Metropolitan Assembly	216.00	51.51	164.49	400.00
Ayawaso Central Municipal	228.00	160.15	67.85	400.00
Ablekuma Central Municipal	260.00	156.54	103.46	400.00
Korle-Klottey Municipal	**303.00**	**641.35**	**-338.35**	400.00
Ayawaso North Municipal	**321.00**	**680.00**	**-359.00**	400.00
Ayawaso East Municipal	**328.00**	**556.66**	**-228.66**	400.00
Ablekuma North Municipal	331.00	157.08	173.92	400.00
Okaikwei North Municipal	**348.00**	**485.72**	**-137.72**	400.00
Ablekuma West Municipal	**391.00**	**589.93**	**-198.93**	400.00
Ga North Municipal	431.00	148.64	282.36	400.00
Ayawaso West Municipal	**479.00**	**1,456.37**	**-977.37**	400.00
La Dade Kotopon Municipal	540.00	239.57	300.43	400.00
Ledzokuku Municipal	**601.00**	**640.41**	**-39.41**	400.00
Krowor Municipal	**675.00**	**1,204.28**	**-529.28**	400.00
Ga Central Municipal	765.00	690.31	74.69	400.00
Tema Metropolitan Assembly	**884.00**	**1,379.13**	**-495.13**	400.00
**Average Urban**	443.81	577.35	**-133.54**	
**Peri-Urban Districts**				
Tema West Municipal	**1,140.00**	**1,824.00**	**-684.00**	400.00
Adentan Municipal	1,268.00	1,142.00	126.00	400.00
La Nkwantanang Madina	**922.00**	**4314.84**	**-3,393.00**	400.00
Municipal				
Ga East Municipal	**674.00**	**1267.27**	**-593.00**	400.00
Ashaiman Municipal	**705.00**	**883.29**	**-178.00**	400.00
Weija Gbawe Municipal	**1,096.00**	**2,840.00**	**-1,744.00**	400.00
Kpone-Katamanso Municipal	**2,307.00**	**4,807.00**	**-2,500.00**	400.00
Ga South Municipal	**2,498.00**	**5,016.00**	**-2,518.00**	400.00
Ga West Municipal	5,521.00	4,811.00	710.00	400.00
**Average Peri-Urban**	**1,792.00**	**2,990.00**	**-1,197.00**	

## Highlights

- The Three-Step Floating Catchment Area (3SFCA) model aided in estimating potential public transport infrastructure and accessibility in a global south city
- Poor transport infrastructure, lack of timely travel information, and poor policy implementation are the major contributing factors to transport inequality in Accra
- There was significant difference in the average distance to a transport access point in different districts in Accra.
- Most areas with low public transport infrastructure availability are often underdeveloped and peri-urban
- An individual's residential location significantly impacts their access to public transport in Accra.
- There is empirical evidence of spatial inequity in public transport infrastructure and services in Accra
